# Sertaconazole 300 mg versus clotrimazole 500 mg vaginal suppository for treating pregnant women with acute vaginal candidiasis: a double-blinded, randomized trial

**DOI:** 10.1186/s12884-024-06440-z

**Published:** 2024-04-04

**Authors:** Chenchit Chayachinda, Manopchai Thamkhantho, Thanapa Rekhawasin, Chanakarn Klerdklinhom

**Affiliations:** 1 Unit of Infectious Diseases, Department of Obstetrics and Gynaecology, Faculty of Medicine Siriraj Hospital, Mahidol University, 2 Wanglang Road, Bangkok Noi, Bangkok, 10700 Thailand; 2 Division of Materno-Fetal Medicine, Department of Obstetrics and Gynaecology, Faculty of Medicine Siriraj Hospital, Mahidol University, Bangkok, Thailand; 3 Department of Nursing, Department of Obstetrics and Gynaecology, Faculty of Medicine Siriraj Hospital, Mahidol University, Bangkok, Thailand

**Keywords:** Clotrimazole; Sertaconazole, Single dose, Pregnancy outcomes, Pregnant women, Vaginal candidiasis

## Abstract

**Background:**

Vaginal candidiasis (VC) commonly affects pregnant women. Traditionally, clotrimazole vaginal tablets (CLO) have been the cornerstone of management. However, sertaconazole ovules (SER) offer a novel topical antimycotic option. This double-blinded, randomized trial evaluated the efficacy of single-dose SER and CLO in treating acute VC during pregnancy.

**Methods:**

From June 2020 to May 2021, this trial recruited pregnant women aged ≥ 18 years with VC symptoms (abnormal vaginal discharge and/or vulvar/vaginal itching) confirmed by microscopy. Participants with ≥ 4 VC episodes in the prior year, immunocompromised status, or imidazole contraindications and those who were absent at the 2-week follow-up were excluded. Participants were randomized to receive either 300 mg SER or 500 mg CLO. Evaluations 2 weeks after the initial medication administration included clinical cure (self-reported resolution of all symptoms), microscopic cure (pseudohyphal absence), patient satisfaction, side effects, and time to clinical cure. Participants with persistent VC received weekly SER doses until delivery. Assessments of recurrence and pregnancy outcomes were done.

**Results:**

The analysis included 96 participants (48 per group, mean age 27.4 ± 7.4 years, gestational age at diagnosis 22.9 ± 6.4 weeks). Without statistical significance, SER achieved a higher clinical cure rate (62.5% vs 50%, *p* = 0.217; a mean difference of 12.5%, 95%CI: -17.5% to 42.5%; and a rate ratio of 1.25, 95%CI: 0.71 to 2.23) and a lower microscopic cure (47.9% vs. 62.5%, *p* = 0.151; a mean difference of -14.6%, 95%CI: -44.3% to 15.1%; and a rate ratio of 0.77, 95%CI: 0.43 to 1.37). The two groups had comparable times to clinical cure (SER: 3.1 ± 1.8 days, CLO: 3.4 ± 2.7 days; *p* = 0.848) and substantial satisfaction rates (SER: 66.7%, CLO: 60.4%; *p* = 0.753). No side effects were reported. Of 60 participants who gave birth at Siriraj Hospital, there were no significant differences in pregnancy outcomes. Repeated SER dosing eradicated symptoms and enhanced the microscopic cure rate. Recurrence was observed in four SER and two CLO participants within 1–2 months.

**Conclusion:**

In the treatment of acute VC during pregnancy, 300 mg SER and 500 mg CLO exhibited comparable efficacy in terms of clinical and microscopic cure rates, satisfaction, side effects, time to clinical cure, recurrence rates, and pregnancy outcomes.

**Trial registration:**

TCTR20190308004 (registration date March 8, 2019).

## Introduction

Vaginal candidiasis (VC), or less frequently referred to as vulvovaginal candidosis, presents a significant gynecologic challenge, compelling affected women to seek medical intervention [1, 2]. Symptoms predominantly include altered discharge exhibiting curd-like properties, augmented volume, and intense vulvar/vaginal itching [3–6]. The principal causative agent, *Candida albicans* [2, 7]*,* resides within the normal vaginal microbiota. Risk factors include a sedentary lifestyle, elevated estrogen levels, stress, and dietary issues, notably iron deficiency anemia and high sugar intake [8, 9]. These conditions render pregnant women particularly vulnerable to VC, with a noted increase in recurrence as pregnancy progresses [2, 10]. Data on the impact of VC on pregnancy outcomes, such as preterm birth and prelabor membrane rupture, remain contentious [11]. Treating VC during pregnancy might offer protective benefits [12].

Oral or vaginal imidazoles serve as the primary treatment for VC [7], with topical formulations recommended for pregnant women. For over four decades, clotrimazole (CLO) vaginal tablets have been a mainstay for treating VC in pregnant women [13], with multiple-dose regimens being favored [9]. Nonetheless, recent systematic reviews suggest that a single 500 mg dose of CLO may offer efficacy comparable to that of multiple lower-strength CLO doses [13]. However, the gravid uterus can complicate vaginal insertion, especially in the third trimester, a peak period for VC recurrence [10]. Additionally, a notable drawback of CLO vaginal tablets is their dissolution time [5]. This limitation led to the introduction of sertaconazole (SER) 300 mg vaginal ovule. This novel formulation dissolves readily at body temperature, sustains a 96-h vaginal presence, and is minimally absorbed systemically [14]. In vitro research has corroborated the antibacterial, anti-inflammatory, and antipruritic effects of SER [15].

Recent studies [3, 5, 6, 16] have shown that SER is noninferior to CLO, with quicker symptom relief and similar adverse effects. Efficacy comparable to that of other topical azoles has also been noted [4, 17]. Nonetheless, the long-term outcomes of SER usage through to delivery have not been thoroughly investigated. This study aimed to evaluate an alternative treatment for VC in pregnant Thai women in both the short and long term. Specifically, we compared the clinical and microscopic cure rates, side effects, patient satisfaction, recurrence, and pregnancy outcomes between patients treated with 300 mg of single-dose SER and those treated with 500 mg of single-dose CLO.

## Materials and methods

### Study design and ethical considerations

This double-blinded, randomized trial was conducted from June 2019 to January 2021 at the Department of Obstetrics and Gynaecology, Faculty of Medicine Siriraj Hospital. In alignment with the Declaration of Helsinki principles, ethical approval was granted by the Siriraj Institutional Review Board (reference: Si-132/2019; registration date: February 11, 2019) prior to initiating participant recruitment. The trial was registered with the Thai Clinical Trial Registry (reference: TCTR20190308004; registration date: March 8, 2019).

### Participants

In our protocol, every woman with abnormal vaginal discharge was subjected to a high vaginal swab for wet mount preparation. The procedure involved mixing the swab with 1 mL of 0.9% normal saline solution and examining it under a light microscope after adding 10% potassium hydroxide solution. Eligible participants were pregnant women aged ≥ 18 years with abnormal discharge or vulvar/vaginal itching, where VC was confirmed by the presence of pseudohyphae, not blastospores, under microscopy. This diagnostic criterion was based on the yeast-to-hyphal transformation characteristic of *C. albicans* pathogenesis [18].

Women who were beyond 36 weeks of gestation or who had a history of ≥ 4 VC episodes in the last year, insulin-dependent diabetes mellitus, human immunodeficiency virus infection, systemic lupus erythematosus, recent use of vaginal suppositories, imidazole allergy, or symptomatic liver disease were excluded. The statistical analysis was limited to participants who returned for the 2-week follow-up.

### Intervention

At the Siriraj Female STD Clinic, pregnant women with abnormal vaginal discharge were briefed about the study during their wait. After comprehensive history-taking and physical, pelvic, and microscopic examinations, study nurses detailed the study to eligible women. Those who fulfilled the eligibility criteria and consented to participate were then randomized at a 1:1 ratio to receive either a CLO 500 mg tablet (Canesten® 500 mg, Bayer, Thailand) or an SER 300 mg vaginal ovule (Zalain®, Pacific Health Care, Thailand). This randomization employed a computer-generated, block-of-four method devised by a statistician.

To ensure blinding, the study drugs were concealed within opaque envelopes and dispensed according to the predetermined block-of-four randomization. Trained study team members, either gynecologists or residents, were responsible for the deep insertion of the medication into the posterior fornix without disclosing the drug to participants. Following the insertion, the participants were instructed to remain in the supine position for 5 min to facilitate drug retention.

Subsequently, participants were interviewed using a case record form, informed about potential local and systemic side effects, and scheduled for a 2-week follow-up by a study nurse. They received guidance on mitigating VC risk behaviors, including avoiding sugar-rich foods and drinks, reducing excessive genital cleansing, refraining from using intimate hygiene products, and avoiding close-fitting garments [8]. Engaging in vaginal intercourse was prohibited during the study. Participants were also asked to track the time until clinical cure of all symptoms. Before the patient left the clinic, expulsion of the vaginal suppository was checked. The entire process on the enrollment day (Visit 0) lasted approximately 40 min.

Outcomes were evaluated at a 2-week follow-up (14 ± 2 days; Visit 1), with a study nurse providing a telephone reminder the day before. At this visit, blinded staff inquired about participants’ symptoms, time to clinical cure, satisfaction levels, and any side effects experienced. A blinded gynecologist conducted pelvic examinations and wet preparations with 10% potassium hydroxide to check for pseudohyphae.

Given the Clinic’s provision of SER at no cost, participants lacking microscopic cure (the absence of pseudohyphae) at Visit 1 received two additional weekly doses of SER and were trained on self-application using a manikin. A follow-up (Visit 2) was scheduled for 2 weeks after the self-administration of the second SER dose. Should pseudohyphae persist at Visit 2, participants were provided with four more weekly SER doses. They were instructed to return 2 weeks following the self-administration of the fourth of these doses (Visit 3) for microscopic assessment. Participants with ongoing pseudohyphae at Visit 3 were prescribed weekly SER doses until delivery.

Participants who achieved microscopic cure were advised to revisit the clinic for any recurrent abnormal vaginal discharge episodes. The pregnancy outcomes of those who gave birth at Siriraj Hospital were documented.

### Outcome measures

The primary endpoint was clinical cure, characterized by the resolution of all initial symptoms, particularly abnormal vaginal discharge and vulvar/vaginal itching. Given the frequent recurrence of vulvovaginal candidiasis during pregnancy and its significant impact on quality of life [19], clinical resolution was a focal concern. Equally critical was microscopic cure, identified by the absence of pseudohyphae in wet preparations with 10% potassium hydroxide. Microscopic cure has been linked to reduced preterm birth rates in asymptomatic patients [12].

The time to clinical cure was defined as the number of days from treatment initiation to the resolution of all symptoms. Participants self-assessed their treatment response on a three-point scale (1 = no/minimal improvement, 2 = moderate improvement, 3 = substantial improvement). The side effects of the study medications were categorized as local (vaginal pain, irritation, swelling) or systemic (skin rash, respiratory distress). Recurrence was defined as a new episode of symptomatic VC, microscopically confirmed by gynecologists, at any time during pregnancy after participants were classified as microscopically cured at Visit 1.

Pregnancy outcomes were documented only for participants who gave birth at Siriraj Hospital. The neonatal outcomes included birth weight and 1- and 5-min Apgar scores. Deliveries prior to 37 gestational weeks were deemed preterm, and a birth weight under 2500 g was classified as low. An Apgar score < 7 at either 1 or 5 min indicated a poor prognosis for the newborn.

### Sample size calculation and statistical analysis

The sample size and statistical analyses were conducted using Stata Statistical Software, release 12.1 (StataCorp LLC, College Station, TX, USA). The calculations were informed by the findings of a study by Lutsevich, which indicated clinical cure rates of 93.4% for SER 300 mg vaginal ovules and 71.9% for a 6-day regimen of CLO 100 mg vaginal tablets among pregnant Russian women with VC [16]. With the aim of achieving 80% power and a significance level of 0.05 (for a two-sample comparison), we determined that each group required 47 participants.

Descriptive statistics are reported as the *n* (%) and mean ± SD. Categorical data comparisons utilized the chi-square test and Fisher’s exact test, while the Shapiro–Wilk test was used to assess continuous data distribution. Parametric data were analyzed using Student’s t test. Binary outcome effect sizes are presented as the mean difference, rate ratio, and 95% CI, with *p* values < 0.05 indicating statistical significance.

## Results

**Fig. 1:**
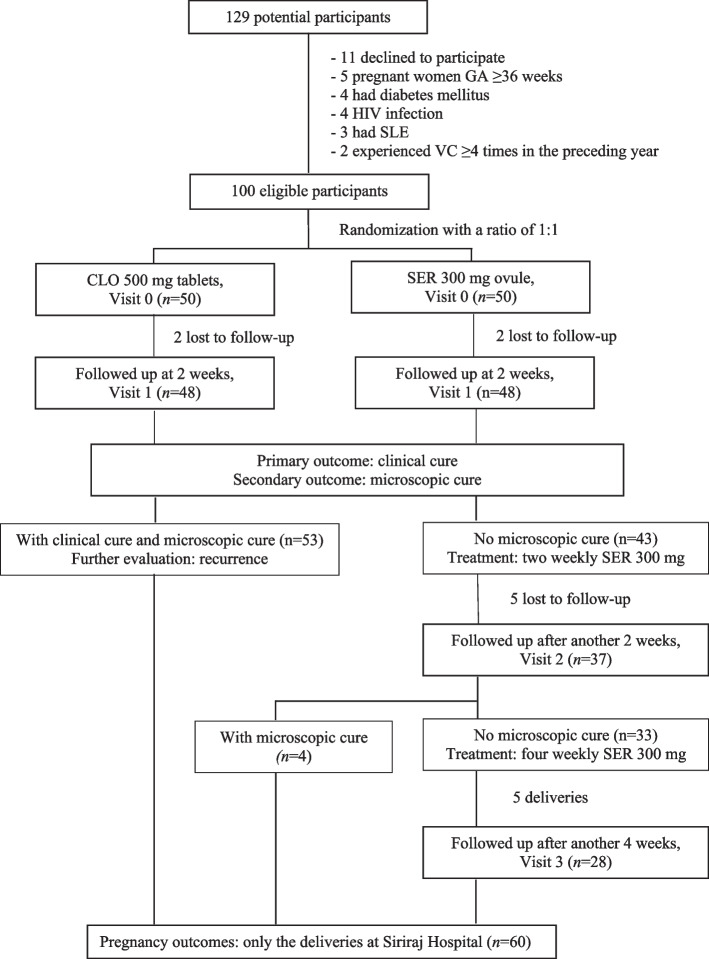
Flow of the participants

Out of 129 potential participants screened, 29 were excluded based on the criteria. Fifty patients were assigned to the CLO group, and another 50 were assigned to the SER group. By Visit 1, two participants from each group were lost to follow-up (Fig. 1), leaving 96 participants for statistical analysis.

As depicted in Table 1, the demographic and clinical characteristics were comparable between the groups. The mean age was 27.4 ± 7.4 years, with a mean body mass index (BMI) of 23.6 ± 5.4 kg/m^2^. Diagnosis occurred at a gestational age of approximately 23 weeks. All participants had abnormal vaginal discharge, and two-thirds experienced vulvar/vaginal itching. Approximately 10% had a history of sexually transmitted infections, comprising anogenital warts (*n* = 8), herpes genitalis (*n* = 2), hepatitis B (*n* = 1), and syphilis (*n* = 1). Almost 40% reported abnormal vaginal discharge in the past month, and 57.3% had experienced 1–2 VC episodes in the previous year.

**Table 1:** Characteristics of the participants (*N* = 96)

**Characteristics**	**Total ( *N* = 96)**	**Clotrimazole (*n*** **= 48)**	**Sertaconazole (*n*** **= 48)**	***p***
Age (y)	27.4 ± 7.4	27.9 ± 7.4	27 ± 7.4	0.565
< 25	44 (45.8)	20 (41.7)	24 (50.0)	0.709
25–35	32 (33.4)	17 (35.4)	15 (31.3)	
> 35	20 (20.8)	11 (22.9)	9 (18.7)	
BMI (kg/m^2^)	23.6 ± 5.4	23.7 ± 6.2	23.4 ± 4.5	0.818
< 23	54 (56.3)	27 (56.3)	27 (56.3)	0.940
23–25	11 (11.4)	6 (12.4)	5 (10.4)	
> 25	31 (32.3)	15 (31.3)	16 (33.3)	
Being parous	25 (26.0)	15 (31.3)	10 (20.8)	0.245
GA at diagnosis (wk)	22.9 ± 6.4	23.0 ± 6.8	22.8 ± 6.1	0.862
≤ 20	37 (38.5)	19 (39.6)	18 (37.5)	0.834
> 20	59 (61.5)	29 (60.4)	30 (62.5)	
Presence of vulvar/vaginal itching	56 (58.3)	30 (62.5)	26 (54.2)	0.408
Abnormal vaginal discharge in prior 4 wk	35 (36.5)	18 (37.5)	17 (35.4)	0.832
Fungal infection in other areas	6 (6.3)	2 (4.2)	4 (8.3)	0.399*
History of STIs	12 (12.5)	7 (14.6)	5 (10.4)	0.537
VC in prior year				0.149
0	41 (42.7)	16 (33.4)	25 (52.0)	
1	19 (19.8)	10 (20.8)	9 (18.8)	
2	36 (37.5)	22 (45.8)	14 (29.2)	

The data are presented as *n* (%), mean ± standard deviation

*BMI* Body mass index, *GA* Gestational age, *STIs* Sexually transmitted infections, *VC* Vaginal candidiasis

^*^Fisher’s exact test

At the first follow-up (Visit 1), compared with CLO recipients, SER recipients exhibited a nominally greater clinical cure rate of 62.5%, with a *p* value of 0.217. This resulted in a mean difference of 12.5% (95% CI: -17.5% to 42.5%) and a rate ratio of 1.25 (95% CI: 0.71 to 2.23). However, the microscopic cure rate was marginally lower at 47.9% for SER versus 62.5% for CLO, with a *p* value of 0.151. This showed a mean difference of -14.6% (95% CI: -44.3% to 15.1%) and a rate ratio of 0.77 (95% CI: 0.43 to 1.37). Neither difference reached statistical significance. The time to clinical cure was similar across groups among those who achieved microscopic cure (3.4 ± 2.7 days for CLO vs. 3.1 ± 1.8 days for SER, *p* = 0.848). Neither group reported any local or systemic side effects. Substantial self-rated satisfaction levels were comparable (60.4% for SER vs. 66.7% for CLO, *p* = 0.753; Table 2).

**Table 2:** Outcome measures at the 2-week follow-up (*N* = 96)

	**Total (*N* = 96)**	**Clotrimazole (*n* = 48)**	**Sertaconazole (*n* = 48)**	***p***
Clinical cure	54 (56.3)	24 (50.0)	30 (62.5)	0.217
Microscopic cure	53 (55.2)	30 (62.5)	23 (47.9)	0.151
Time to clinical cure (d)	3.2 ± 2.4	3.1 ± 1.8	3.4 ± 2.7	0.848
Self-rated satisfaction				
No/Minimal	12 (12.5)	6 (12.5)	6 (12.5)	0.753
Moderate	23 (24.0)	10 (20.8)	13 (27.1)	
Substantial	61 (63.5)	32 (66.7)	29 (60.4)	

The data are presented as the *n* (%), mean ± standard deviation

Among the participants who achieved microscopic cure at the initial follow-up, four patients in the SER group experienced recurrence within 1 to 2 months, while two patients in the CLO group experienced recurrence at 2 months. Among those without initial microscopic cure, 4/37 (10.8%) achieved it within the following 2 weeks. Of the 33 participants who continued without microscopic cure, 28 attended the subsequent follow-up, with 6 (21.4%) achieving cure.

Pregnancy outcomes did not significantly differ between the CLO and SER groups (Table 3). All instances of preterm birth were post-34 weeks gestation, and infants classified as having low birth weights all exceeded 2200 g. Two newborns with initial Apgar scores below 7 improved following brief oxygen therapy.

**Table 3:** Pregnancy outcomes (*N* = 60)

	**Total (*N* = 60)**	**Clotrimazole (*n* = 30)**	**Sertaconazole (*n* = 30)**	***p***
GA at delivery (wk)	37.9 ± 1.9	37.9 ± 2.2	37.9 ± 1.5	0.942
Preterm birth	7 (11.7)	3 (9.7)	4 (13.3)	0.654
Birth weight (gm)	2954 ± 421	2960 ± 491	2904 ± 353	0.614
Low birth weight	6 (10)	2 (6.7)	4 (13.3)	0.389*
Apgar score < 7 at 1 or 5 min	2 (3.3)	2 (6.7)	0	0.150*

The data are presented as *n* (%), mean ± standard deviation

GA, gestational age

^*^Fisher’s exact test

## Discussion

This study is the first direct comparison of two single-dose vaginal antimycotic suppositories for treating VC in pregnant women. Echoing findings from a previous study of pregnant Russian women treated with a single SER 300 mg dose versus a 7-day CLO 100 mg regimen [16], SER achieved a 12.5% higher clinical cure rate than than that of CLO, but the difference was not statistically significant. The onset of symptom improvement was similar, occurring at approximately three days. In contrast, earlier research comparing the older formulation of SER 500 mg vaginal tablet to multiple lower-strength CLO doses presented mixed outcomes. Specifically, two studies involving nonpregnant Indian women [3, 6] noted better outcomes with SER, whereas a study involving nonpregnant Thai women [5] observed higher cure rates with CLO and reported notably lower overall cure rates. This issue warrants further exploration, as fungal culture was not performed in the present study.

The increased colonization of *Candida albicans*, elevated estrogen levels, altered immune function, and disrupted glucose metabolism during pregnancy [20] justify the extended regimen of topical VC treatments for expectant mothers [9, 20]. Nevertheless, administering multiple intravaginal doses can be impractical for those with a gravid uterus, particularly in the third trimester, a time at which a heightened recurrence risk is noted [10]. Additionally, the practice of intravaginal insertion can be particularly challenging for Asian populations, including Thais [21]. A prior study among pregnant Russian women, who were treated twice with one vaginal suppository of 500 mg SER every 7 days, showed a 90% cure rate [22] which was comparable to that of multiple-dose clotrimazole regimens in pregnant women of various ethnicities [13], our study revealed improved outcomes with added weekly doses of 300 mg SER ovules. However, our investigation lacked a crossover design and a no-treatment control group, highlighting the need for further research to substantiate the efficacy of repeated single-dose antimycotic vaginal suppositories.

Concerns about the safety of SER during pregnancy have been notable. With more than 45 years of use and extensive evidence supporting its safety in pregnancy (category B) [13], CLO sets a high standard. Sertaconazole, an imidazole similar to CLO, has shown minimal systemic absorption in in vitro studies [14]. The current investigation corroborates findings from a large-scale French study involving 16 222 pregnant women exposed to SER and 91 976 controls, indicating no significant difference in the risk of congenital anomalies and adverse pregnancy outcomes [23].

The impact of VC on the vaginal environment can contribute to adverse pregnancy outcomes [11], including a marginal increase in preterm births among untreated symptomatic women [24]. Although evidence is sparse, SER is known for its anti-inflammatory effects and ability to combat *Gardnerella* spp., a bacterial vaginosis-associated bacterium [25]. Despite the careful monitoring of participants in the current study until delivery, the incidence of preterm births was consistent with the general rates observed in the broader population at our facility [26]. This finding parallels the suspected association between bacterial vaginosis and preterm birth, where inflammation might commence before intervention [27]. A recent systematic review and meta-analysis highlighted the lack of conclusive data supporting routine vulvovaginal candidiasis screening in pregnant women [28].

Despite recurrence typically being more prevalent during late gestation [10], our study noted a low recurrence rate. Unlike prior research [5, 6], we identified only six instances of recurrence. Our methodology might have contributed to this variance. Earlier studies assessed cure rates 1 week after the SER dose and identified recurrences at 4–6 weeks. However, our analysis was conducted during Visit 1, which was 2 weeks after the initial SER dose. Additionally, our analysis only recognized recurrences among participants who had achieved microscopic cure by Visit 1. Another explanation for the differences between our study results and those of earlier studies is that the influence of lifestyle modifications on treatment outcomes was emphasized for all of our participants [7]. Generally recognized lifestyle changes include avoiding sugar intake, stress, and close-fitting clothing; abstaining from sexual activity during treatment; and limiting genital cleansing [8]. Furthermore, providing information on the nature of VC and encouraging open discussion can alleviate participants’ anxiety and reinforce lifestyle modifications [8]. We encourage all healthcare settings to provide comprehensive counseling in addition to standard treatment.

The study’s primary strength lies in its double-blinded, randomized design, ensuring that participants and outcome evaluators were blinded. Furthermore, the inaugural head-to-head comparison of two single-dose azole treatments in pregnant women is important. Both study drugs required a single administration and were properly applied by a well-trained study team member. However, the limitations of this study include the absence of fungal cultures and the lack of a control group for following up patients who lacked microscopic cure at Visit 1. Fungal cultures would provide deeper insights into the modest treatment efficacy observed, especially given the rising global concern over antifungal resistance. Moreover, while the presence or absence of microscopic cure signals a disrupted vaginal ecosystem, treatment based on this finding remains controversial.

## Conclusions

In summary, patients with SER exhibited a greater clinical cure rate but a lower microscopic cure rate than patients with CLO; however, neither of these differences reached statistical significance. Both treatments showed comparable times to clinical cure, levels of self-rated satisfaction, side effect profiles, and pregnancy outcomes. Future research involving larger cohorts is essential to fully ascertain the clinical efficacy and safety of SER for treating VC in both pregnant and nonpregnant populations.

## Data Availability

The study’s dataset is accessible as part of the submission process and is available for public access. For inquiries, contact Associate Professor Chenchit Chayachinda at chenchit.cha@mahidol.ac.th.

## Abbreviations

CLO: Clotrimazole
SER: Sertaconazole
VC: Vaginal candidiasis
